# Spontaneously Corrected Hypoplastic Left Heart: A Case Report and Exceptional Opportunity to Discuss Etiology with Novel Therapeutic Vision

**DOI:** 10.34172/aim.31185

**Published:** 2024-09-01

**Authors:** Mohsen Shahidi

**Affiliations:** Department of Pediatric Cardiology, Besat Hospital, Kurdistan University of Medical Sciences, Sanandaj, Iran

**Keywords:** Early intervention, Foramen oval, Left heart hypoplasia syndrome, Prenatal ultrasound, Ventricular septal defect

## Abstract

Hypoplastic left heart syndrome (HLHS) is a relatively prevalent fetal echocardiography finding in complex congenital heart diseases. Current studies indicate that intrinsic and extrinsic mechanisms could be involved in the development of left heart hypoplasia. Left ventricular inflow or outflow disorders may cause left heart hypoplasia. Prenatal aortic valvuloplasty has become more common as a therapeutic strategy. Our case presentation provides an opportunity for a new vision toward the etiology, prevention, and treatment of HLHS. In our patient, prenatal progressive left heart hypoplasia associated with restrictive foramen oval (FO) suggested the likelihood of a flow-mediated mechanism. Additionally, postnatal improvement of the hypoplastic left heart in the presence of a functional perimembranous ventricular septal defect (PM-VSD) reinforced the suspicion of an extrinsic mechanism. Pre- or postnatal interventional creation of an atrial septal defect (ASD) or VSD is our proposed method for HLHS in selected patients.

## Introduction

 Hypoplastic left heart syndrome (HLHS) occurs in 1% to 3.8% of all congenital heart diseases.^1^ The severity of left ventricular hypoplasia is usually interrelated with the degree of valvar hypoplasia, so-called the hypoplastic left heart complex.^2^ The degree of valvar stenosis or atresia is defined as mitral stenosis/aortic stenosis; mitral stenosis/aortic atresia; mitral atresia/aortic atresia.^3^ In severe cases of HLHS, the right ventricle (RV) is the dominant chamber, supplying both systemic and pulmonary blood circulation with ductus arteriosus-dependent systemic flow.^1,4^ It is usually associated with poor prognosis and accounts for approximately 25% of cardiac deaths within the first year of life.^5,6^ Norwood operation is the proposed surgery for these cases, recognized as the reconstruction of the ascending aorta and aortic arch during the first stage, followed by the succeeding two palliative operations (Glenn and Fontan).^7^

 There is no definite consensus about the proposed mechanisms of HLHS and its embryologic process.^1^ The extrinsic mechanism advocates “irreversible flow-mediated cardiac damage” resulting in persistent disease despite improvement of normal flow.^6,8^ On the other hand, the intrinsic theory insists on the primary defect in myocardial development.^6^ Chromosomal abnormality and single or multiple-gene defects account for about one-quarter of HLHS cases.^1,3^ Furthermore, fetal exposure to teratogens and active maternal infections, like herpesvirus, coxsackievirus, and cytomegalovirus, may be a risk for HLHS.^1^

## Case Report

**Figure 1:**
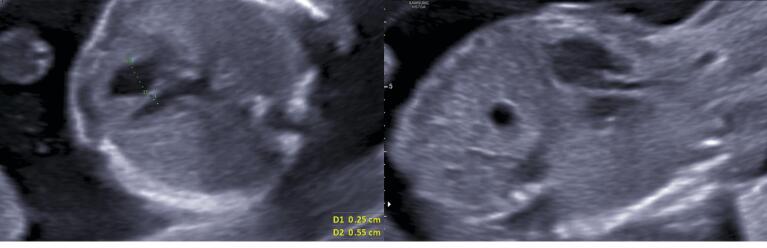
Prenatal Axial and Sagittal Views of a 2-Dimensional Heart Ultrasound Demonstrating Left Ventricular and Aortic Hypoplasia in an 18-Week Fetus

 A 28-year-old pregnant woman gravid 1/para 0 whose gestational age was 18 weeks with a singleton female referred for fetal echocardiography. The echocardiographic evaluation revealed a left ventricle (LV) smaller than the RV in the four-chamber view (with a maximum dimension of 2.5 mm for the LV versus 5.5 mm for the RV) and relatively small aortic and mitral valves (AV and MV: 1.9 mm and 2.5 mm, respectively) (Figure 1). There was a retrograde flow through the aortic arch, but the ductal arch had a complete ante-grade flow. The foramen oval (FO) was restrictive, demonstrated by turbulent color flow through the small orifice estimated to be approximately 1.5 mm. Likewise, pulmonary venous spectral Doppler showed an increased S/D wave ratio and prominent reversal A wave.

**Figure 2:**
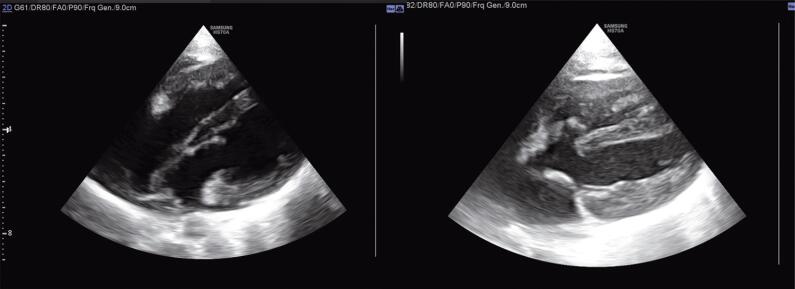
Four and Five Chamber Views of Trans-thoracic 2-Dimensional Echocardiography in a 9 Months Infant with Self-corrected Left Ventricular Hypoplasia and Pouch-Shaped Perimembranous Ventricular Septal Defect. (Left Ventricle is the lower chamber)

 The patient’s family insisted on the continuation of pregnancy. The second echocardiographic assessment at 28 weeks of gestational age revealed similar results. The baby had a comfortable delivery without respiratory distress or hemodynamic threat during the few days of NICU care. The first postnatal echocardiography showed severe left heart hypoplasia with a small rudimentary LV (max LV dimension at four-chamber view = 3.2 mm vs RV = 11 mm). The mitral and aortic valves were anatomically visible but had small diameters (MV = 4 mm, AV = 3 mm). The FO seemed to be closed with no visible shunt. Surprisingly, for the first time, an aneurysmal pouch-shaped perimembranous ventricular septal defect (PM-VSD) (RV side orifice = 2.5 mm) was identified with a visible left to right shunt (QP/QS = 1.38). The ascending aorta was narrow at the origin (3 mm) but with an acceptable size at the aortic arch (4.7 mm). The ductus arteriosus was patent with a relatively large size (2.2 mm) and a retrograde color flow toward the aortic arch. Pulmonary pressure was in the normal range. She was discharged and referred to a professional heart center. Their new assessment confirmed the previous diagnosis. The re-evaluation of the 9-month-old patient yielded unbelievable echocardiographic findings. The size of the LV chamber was appropriate (max LV diameter = 6.3 cm vs RV = 6 cm) with acceptable left ventricular inflow and outflow tracts (Figure 2). The mitral and tricuspid valve annuluses had relatively similar sizes (MV = 1.5 cm vs TV = 1.8 cm). The PM-VSD was the same size and had a left-to-right shunt (Figure 2). The ascending aorta also had an appropriate diameter (7 mm) with normal forward flow. The ductus arteriosus was closed. She had a good appetite with satisfactory growth and development. Subsequent echocardiographic evaluations were optimal with regular pulmonary arterial pressure but the presence of a small PM-VSD.

## Discussion

 Mechanical flow disorders are some of the proposed theories in HLHS evolution.^8^ On the other hand, intrinsic cardiomyocyte disorders, including molecular and genetic factors, might coincide or act as a sole etiology.^3,5,9,10^

 In our patient, the restrictive FO was associated with the progressive evolution of left heart hypoplasia during the fetal period, indicating the likelihood of a flow-mediated mechanism. Alex Veldman et al reported the development of the HLHS phenotype in sheep fetal hearts after FO occlusion within 3-4 weeks.^11^ Rahman et al described the evolution of LV and aortic hypoplasia through surgical ligation of the mitral valve in fetal lambs.^8^ Another investigation was conducted by left atrial ligation on embryonic chicks with similar results.^12^ On the other hand, the congenital oblique orientation of FO accounted as a pathophysiologic mechanism of HLHS due to the hindering of easy flow from the inferior vena cava and right atrium toward the left chambers.^4^ Likewise, the observation of small or absent FO in 6%‒22% of HLHS patients reinforces the substantial causative role of the restrictive FO.^4^

 Postnatal improvement of left cardiac hypoplasia reinforced the primary suspicion of a restrictive flow mechanism in our patient. Theoretically, postnatal enhancement of pulmonary venous flow toward the LA and LV resulted in optimal left ventricular circulation in the presence of a left-to-right shunt through the small PM-VSD. We missed the PM-VSD during the prenatal period, probably due to the absence of a shunt and functional closure of the aneurysmal sac by increased RV pressure. Supposedly, higher LV pressure after birth was associated with a functional PM-VSD. Hattam A T. reported that HLHS will not develop in the presence of functional VSDs due to the preservation of enough flow toward the LV despite the inflow or outflow obstruction.^13^ HLHS is not typically associated with other cardiac anomalies. The association of HLHS and VSD is a rare phenomenon.^8^ Consequently, prenatal VSD creation is considered a possible therapeutic method of left ventricular rescue.^13^

 We assumed that the postnatal functional PM VSD was the milestone for both LA decompression and sufficient flow through the left chambers in our patient. On the other hand, the postnatal rescue of the left-sided heart might be partially owing to the later prenatal initiation time of the inflow disorder.^8^ Habereret al suggested possible predictors of future biventricular physiology in fetuses with HLHC.^14^

 In addition to flow-mediated HLHS (extrinsic factors), intrinsic factors such as apoptosis may contribute to the development of HLHS.^10^ The prolonged administration of human induced pluripotent stem cells from patients with HLHS advocated intrinsic cardiomyocyte disorders.^4^ Furthermore, genetic or environmental interruption of the Ca^2+^ signalling pathway and mutations in Rbfox2 may disturb cardiac chambers, outflow tracts, and valves.^6,9^

## Conclusion

 Considering restrictive FO as a predisposing factor for left cardiac hypoplasia provokes the idea of prenatal atrial septostomy. Likewise, pre- or postnatal creation of ventricular septal defects in selected HLHS patients may increase left heart circulation and improve hypoplasia. Serial fetal echocardiography evaluation, including estimating the starting time of left heart hypoplasia, quality of progression, fluency of FO, color and spectral Doppler assessment of pulmonary venous flow, Z scores of the LV chamber and its inflow/outflow tracts and the presence or absence of EFE may provide appropriate case selection for these procedures.^14^ However, further investigations are required to support the reliability of the proposed interventions.
